# Tumor Cell Extrinsic Synaptogyrin 3 Expression as a Diagnostic and Prognostic Biomarker in Head and Neck Cancer

**DOI:** 10.1158/2767-9764.CRC-21-0135

**Published:** 2022-09-15

**Authors:** Ryan M. Murphy, Jason Tasoulas, Alessandro Porrello, Miranda B. Carper, Yi-Hsuan Tsai, Alisha R. Coffey, Sunil Kumar, Peter YF. Zeng, Travis P. Schrank, Bentley R. Midkiff, Stephanie Cohen, Ashley H. Salazar, Michele C. Hayward, D. Neil Hayes, Andrew Olshan, Gaorav P. Gupta, Anthony C. Nichols, Wendell G. Yarbrough, Chad V. Pecot, Antonio L. Amelio

**Affiliations:** 1 Graduate Curriculum in Pharmacology, University of North Carolina at Chapel Hill, Chapel Hill, North Carolina.; 2 Division of Oral and Craniofacial Health Sciences, Adams School of Dentistry, University of North Carolina at Chapel Hill, Chapel Hill, North Carolina.; 3 Lineberger Comprehensive Cancer Center, University of North Carolina at Chapel Hill, Chapel Hill, North Carolina.; 4 Bioinformatics Core, Lineberger Comprehensive Cancer Center, UNC School of Medicine, The University of North Carolina at Chapel Hill, Chapel Hill, North Carolina.; 5 Technology Development, Naveris Inc., Natick, Massachusetts.; 6 Department of Otolaryngology - Head and Neck Surgery, University of Western Ontario, London, Ontario, Canada.; 7 Department of Pathology and Laboratory Medicine, University of Western Ontario, London, Ontario, Canada.; 8 Department of Otolaryngology/Head and Neck Surgery, University of North Carolina School of Medicine, Chapel Hill, North Carolina.; 9 Pathology Services Core, Lineberger Comprehensive Cancer Center, UNC School of Medicine, The University of North Carolina at Chapel Hill, Chapel Hill, North Carolina.; 10 Center for Cancer Research, University of Tennessee Health Sciences, Memphis, Tennessee.; 11 Department of Epidemiology, University of North Carolina at Chapel Hill, Chapel Hill, North Carolina.; 12 Department of Radiation Oncology, UNC School of Medicine, Chapel Hill, North Carolina.; 13 Department of Oncology, University of Western Ontario, London, Ontario, Canada.; 14 Department of Pathology and Lab Medicine, University of North Carolina School of Medicine, Chapel Hill, North Carolina.; 15 Department of Medicine, University of North Carolina at Chapel Hill, Chapel Hill, North Carolina.; 16 Department of Cell Biology and Physiology, School of Medicine, University of North Carolina at Chapel Hill, Chapel Hill, North Carolina.; 17 Cancer Cell Biology Program, Lineberger Comprehensive Cancer Center, School of Medicine, University of North Carolina at Chapel Hill, Chapel Hill, North Carolina.

## Abstract

Over 70% of oropharyngeal head and neck squamous cell carcinoma (HNSC) cases in the United States are positive for human papillomavirus (HPV) yet biomarkers for stratifying oropharyngeal HNSC patient risk are limited. We used immunogenomics to identify differentially expressed genes in immune cells of HPV(+) and HPV(−) squamous carcinomas. Candidate genes were tested in clinical specimens using both qRT-PCR and IHC and validated by IHC using the Carolina Head and Neck Cancer Study tissue microarray of HNSC cases. We performed multiplex immunofluorescent staining to confirm expression within the immune cells of HPV(+) tumors, ROC curve analyses, and assessed survival outcomes. The neuronal gene *Synaptogyrin-3* (*SYNGR3*) is robustly expressed in immune cells of HPV(+) squamous cancers. Multiplex immunostaining and single-cell RNA sequencing analyses confirmed SYNGR3 expression in T cells, but also unexpectedly in B cells of HPV(+) tumors. ROC curve analyses revealed that combining SYNGR3 and p16 provides more sensitivity and specificity for HPV detection compared with p16 IHC alone. Patients with SYNGR3-high HNSC have significantly better prognosis with 5-year OS and DSS rates of 60% and 71%, respectively. Moreover, combining p16 localization and SYNGR3 expression can further risk stratify HPV(+) patients such that high cytoplasmic, low nuclear p16 do significantly worse (HR, 8.6; *P* = 0.032) compared with patients with high cytoplasmic, high nuclear p16. SYNGR3 expression in T and B cells is associated with HPV status and enhanced survival outcomes of patients with HNSC.

**Significance:**

These findings indicate that codetection of SYNGR3 in immune cells and p16 in tumor cells by IHC can more reliably identify the HPV(+) subgroup of patients with low-risk head and neck cancer that may be appropriate for clinical trials involving treatment deescalation.

## Introduction

Head and neck squamous cell carcinomas (HNSC) are the sixth most common cancer worldwide (1–4). Patients with HNSC are frequently treated with combinatorial therapy consisting of surgery and adjuvant radiation or chemoradiation, or upfront chemoradiation. Radiation treatment–associated morbidities, including loss of taste, reduced salivary flow, and swallowing dysfunction, can be permanent and can substantially impact patient quality of life (5, 6). Multiple genomic and transcriptomic studies have profiled HNSCs and identified distinct molecular subtypes that can be grouped in part based on human papilloma virus (HPV) status (7–9). In fact, knowledge of HPV status has revolutionized the field of head and neck oncology and is now starting to impact treatment planning given that patients with HPV(+) HNSC have distinct clinicopathologic features with significantly better prognosis compared with their HPV(−) counterparts (10–20). Correctly classifying HPV(+) HNSC, as well as which of these patients have improved prognosis and therefore may benefit from treatment deescalation is a major focus of the oncology community with the overarching goal of limiting the severity of treatment-induced side effects without reducing treatment efficacy (21–28). Unfortunately, the incidence and proportion of HPV(+) HNSCs is rising (29–33) further emphasizing the need to correctly identify patients with HPV-associated disease.

Among the currently available HPV detection assays commonly used in clinical settings are those involving PCR of the viral oncogenes E6/E7, IHC of p16 protein, and ISH of either DNA or RNA for E6/E7. These assays have varying reliability and availability depending upon sample requirements, test specificity and sensitivity, as well as presence of specific equipment and technical expertise (29). While PCR-based detection methods of active HPV transcription are both sensitive and highly specific (29–33) and the FDA approved PCR to detect E6/E7 mRNA as the “gold standard” several years ago (34), widespread implementation of this approach has proven difficult. This can be attributed to several technical challenges (35–37), including dependence on fresh-frozen tumor tissue and in distinguishing between false negatives (e.g., arising from improper sample collection, specimen degradation, presence of PCR inhibitors) and false positives (e.g., detecting sample contaminants) making this approach impractical for routine use in many clinical locations (30, 38). In contrast, IHC detection of p16 overexpression in formalin-fixed paraffin-embedded (FFPE) tissues has proven to be globally more accessible and far more reliable because it affords high sensitivity in HPV detection. Unfortunately, p16 IHC lacks specificity and is prone to false positives due in part to accumulating evidence demonstrating that a subset of HPV(−) HNSCs overexpress p16 (39). Consequently, PCR-based assays and p16 IHC remain suboptimal in their performance when used alone for the detection of HPV (26, 29, 39–45). The combined application of ISH to detect expression of the viral E6/E7 genes can overcome sensitivity limitations of p16 IHC but this method is prone to false negatives and is not universally available (46–52). Although IHC and ISH can be multiplexed in many laboratories (53), IHC is a relatively inexpensive and more standard assay for pathology laboratories leading many clinicians to rely on p16 IHC alone for classification of these tumors. Therefore, there is a significant unmet need to identify additional clinically useful biomarkers that suitable for multiplexed IHC on the same sample slide that provide accurate detection of HPV status to aid in stratifying patient risk better.

In addition to identifying an affordable and easily implemented HPV biomarker to complement p16 IHC, the head and neck oncology field is also in need of identifying markers that can predict response to standard and immune-based therapies as a priority. The introduction and increasing popularity of immune-based therapies for various cancers, has provided a viable treatment option for some patients, but only a small proportion of patients with HNSC (15%–20%) respond to these therapies (54–56). Recent studies unveiling the diversity of tumor-immune microenvironment in HNSC present an abundance of opportunities to further examine the roles of these interactions (57–63). Importantly, HPV(+) tumors possess a unique tumor-immune landscape, including differing types, proportions, and functions of immune cells when compared with HPV(−) tumors and recent studies demonstrate these HPV(+) tumors harbor functional PD-1^+^TCF-1^+^CD45RO^+^ stem-like CD8 T cells suggesting that these patients with HNSC retain the ability to respond to PD-1 checkpoint blockade (64–68). These insights led us to take an immunogenomic approach to identify a more reliable HPV biomarker for diagnostic use. In our study, we utilized The Cancer Genome Atlas (TCGA) RNA sequencing (RNA-seq) data (68) to bioinformatically identify differentially expressed genes (DEG) within immune cells of HPV(+) versus HPV(−) HNSCs. Surprisingly, we identified a neuronal synaptic gene, Synaptogyrin-3 (SYNGR3; refs. 69, 70), as a top DEG in immune cells of HPV(+) tumors and association of this novel biomarker with significantly increased 5- and 10-year overall survival (OS) and disease-specific survival (DSS).

## Materials and Methods

### Clinical Samples

All research involving human tumor tissues was reviewed and approved by The University of North Carolina at Chapel Hill Institutional Review Board (IRB) under IRB protocols 15-1604 and 17-2947 and the studies were performed in accordance with recognized U.S. Common Rule ethical guidelines. We obtained a waiver of written informed consent from all subjects for the use of their biological specimens. Fresh-frozen HNSC human tumor specimens with affiliated HPV assay clinical diagnoses were obtained through NC Cancer Hospital and UNC Lineberger Comprehensive Cancer Center's Pathology Services Core. Samples (total *n* = 11) were from the following anatomic sites: larynx (*n* = 2), oral cavity (*n* = 7), and oropharynx (*n* = 2). Histopathologic assessments were made by a pathologist and presented in a nonquantitative, binary format (either negative or positive) for both HPV ISH (high-risk HPV strains) and p16 IHC.

### Bioinformatics

#### Bulk RNA-seq Analysis

TCGA RNA-seq datasets used in this study were downloaded from The Broad Institute TCGA GDAC Firehose (gdac.broadinsitue.org), which provides TCGA level 3 data and level 4 analyses packaged in a form amenable to immediate algorithmic analysis. Specifically, publicly available HNSC tumor data from TCGA were used to evaluate the differential expression of genes between HPV(+) and HPV(−) subjects. HPV(+) samples were defined as having a gene expression–based ratio *E6/E7* > 0 (*n* = 53), whereas HPV(−) samples were required to have a negative HPV status as determined by both ISH and by p16 IHC testing (*n* = 56). Normal samples were omitted from the analysis, according to TCGA records (clin.merged file). Similarly, the cervical squamous cell carcinoma and endocervical adenocarcinoma (CESC) tumor data from TCGA was also used to perform differential expression analysis between HPV(+) (*n* = 281) and HPV(−) (*n* = 22) subjects. HPV status was taken from the available patient HPV test results column in the available merged clinical data. Normal samples were also omitted. Differential expression analysis on both individual bulk RNA-seq datasets was performed using the DESeq2 package in R (DESeq, RRID:SCR_000154; ref. 71). DEGs were defined as having an adjusted *P* value < 0.05, absolute value of log_2_ fold change > 1, and baseMean > 10. Heatmaps were generated using the ComplexHeatmap package in R (ComplexHeatmap, RRID:SCR_017270; ref. 72). The GSE65858 dataset for the oropharyngeal squamous cell carcinoma (OPSCC) expression profiles was downloaded from the Gene Expression Omnibus (GEO) database (https://www.ncbi.nlm.nih.gov/geo/), and transcript abundance was normalized using quantile normalization. Abundance differences were assessed using Wilcoxon signed-rank test.

#### Modified “immunome” Signature

The analyzed immune-related genes were previously identified as belonging to the modified “Immunome” signature’, which can be found in Porrello and colleagues (73). In particular, genes had to belong to the core portion of this collection of gene sets, which is made up of the following 26 immune cell types: activated dendritic cells, B cells, CD8 T cells, cytotoxic cells, dendritic cells (DC), eosinophils, immature DCs, macrophages, mast cells, neutrophils, natural killer (NK) CD56 Bright, NK CD56 Dim, NK Cells, plasmacytoid DCs, T cells, Th cells, T (lymphocyte) central memory, T (lymphocyte) effector memory, T (lymphocyte) follicular helper, T (lymphocyte) gamma delta, T helper (type) 1 (Th1), T helper 17 (Th17), T helper (type) 2 (Th2), regulatory T cells, immune checkpoints [namely, CD274 (PDL1), CTLA4, and PDCD1 (PD1)], and myeloid-derived suppressor cells. Genes belonging to these 26 signatures (577 genes) were included in the summary heatmaps only when supported by statistical evidence (FDR < 0.05) of being differentially expressed in HPV(+) relative to HPV(−) in either bulk RNA-seq dataset.

#### Single-cell RNA-seq Analyses

Publicly available HNSC single-cell RNA-seq data were used to evaluate *SYNGR3* expression level in various immune cell types across HPV-positive (*n* = 8) and HPV-negative (*n* = 18) samples (57, 68). Single-cell RNA-seq analysis was performed using the Seurat v4 package in R (74). Potential doublets and dying cells were filtered out requiring each cell to have less than 6,000 unique features, less than 50,000 mRNAs, and less than 25% mitochondrial gene counts. These filtering criteria resulted in 21,057 cells from the HPV(+) subjects, and 39,919 cells from the HPV(−) subjects. Cell clusters were annotated using the SingleR package (75) using the Monaco immune cell type reference (76).

### RNA Isolation and Real-time qPCR

Tissues were homogenized as described previously (77). NucleoZOL (Macherey-Nagel, catalog no.: 740404.200) was used in accordance with the manufacturer's instructions to extract RNA from fresh-frozen human HNSC tumors. iScript cDNA Synthesis Kit (Bio-Rad, #1708890) was used to make cDNA from extracted RNA. FastStart Universal SYBR Green Master (Rox) Mix (Roche, catalog no.: 04913850001) was used with 1/20 volume of cDNA iScript reaction and 0.25 μmol/L primers. Primer sequences are listed in Supplementary Table S1. Relative gene expression was determined using the 2^ΔΔ^*^C^*_t_ method and normalized using human and mouse RPL23.

### Tissue Microarray

The Carolina Head and Neck Cancer Study (CHANCE) tissue microarray (TMA) used for these studies includes distinct anatomic locations of the oropharynx, hypopharynx, oral cavity, and larynx and has been thoroughly characterized previously (78). Slides used were reviewed for presence of evaluable tumor. Cores lacking evaluable tumor or with fewer than 500 cells detected by analysis algorithm were excluded. Data presented here include 190 evaluable tumor cores taken from 98 separate tumors (1–3 cores/tumor block). Patient details including sex, race/ethnicity, smoking status, pack years, alcohol use, and diagnosis age are described in Supplementary Table S2.

### HPV ISH

Ventana Benchmark XT autostainer was used for HPV ISH according to manufacturer's protocol as described previously (79). INFORM HPV III Family 16 Probe (B, Ventana Medical Systems) was used for staining of HPV strains 16, 18, 31, 33, 35, 39, 45, 51,52, 56, 58, and 66. Either punctate or diffuse signal pattern in tumor nuclei was scored as positive staining. HPV ISH positive was defined as nuclear score 1–3 at any percent.

### Antibodies

Anti-SYNGR3 rabbit polyclonal antibody (referenced as Antibody #1) was purchased from Invitrogen (PA5-60146, Lot182031, RRID:AB_2648137, Thermo Fisher Scientific). Anti-SYNGR3 (E-11) mouse mAb (referenced as Antibody #2) was purchased from Santa Cruz Biotechnology (sc-271046, LotI1718, RRID:AB_10611955, Santa Cruz Biotechnology, Inc.). Anti-p16 mouse mAb (D-25) used for individual stain was Sigma-Aldrich (MAB4133, RRID:AB_95069, Chemicon International Company/Millipore Corporation); for multiplex staining anti-p16 mouse mAb by Ventana (705-4793, Lot Y01733, RRID:AB_2833232, Ventana Medical) was used. pan-Cytokeratin rabbit polyclonal antibody used was from Dako (Z0622, RRID:AB_2650434, Agilent Technologies). A mouse mAb to CD3 (NCL-L-CD3-565, Lot6055982, RRID:AB_563541) and a CD45 mouse mAb (PA0042, Lot66010, RRID:AB_442104) from Leica (Leica Microsystems Inc.) were used for multiplex staining.

### IHC

Chromogenic IHC was performed on paraffin-embedded tissues that were sectioned at 5 μm. All IHC was carried out in the Bond III Autostainer (Leica Microsystems Inc.). Slides were dewaxed in Bond Dewax solution (AR9222) and hydrated in Bond Wash solution (AR9590). Antigen retrieval was performed for 20 minutes at 100°C in Bond-Epitope Retrieval solution 1, pH-6.0 (AR9961).

#### Individual Stains

For the SYNGR3 Invitrogen antibody, slides were incubated for at 1:500 for 1 hour then ImmPress horseradish peroxidase (HRP) anti-rabbit IgG secondary (MP-7451, RRID:AB_2631198, Vector Laboratories). For the SYNGR3 Santa Cruz antibody, slides were incubated at 1:50 for 4 hours followed by Novocastra Post Primary (Leica, #RE7159) and Novolink Polymer (Leica, #RE7161) secondary antibodies for 8 minutes each. Antibody detection with 3,3′-diaminobenzidine (DAB) was performed using the Bond Intense R detection system (DS9263) with ImmPress HRP anti-rabbit IgG (MP-7451, RRID:AB_2631198, Vector Laboratories).

#### Multiplex Stains

Slides were incubated with SYNGR3 Invitrogen antibody at 1:300 for 1 hour and were detected with ImmPress HRP anti-rabbit IgG and TSA Cy5 (SAT705A001EA). After completion of SYNGR3 staining, a second round of denaturation (10 minutes, Bond-Epitope Retrieval solution 1) was followed by incubation in either anti- pan-Cytokeratin (30 minutes, 1:1,500) or CD45 (30 minutes; ready to use) and detection with ImmPress HRP anti-rabbit IgG and TSA Cy3 (SAT704A001EA; Perkin Elmer). Following pan-Cytokeratin/CD45 staining, a third denaturation step was performed for 10 minutes in Bond-Epitope Retrieval solution 2 (pH 9.0; AR9640) followed by incubation with either anti-CD3 (1 hour, 1:200) or p16 (1 hour, 1:5) then detection with Bond Polymer (DS9455) and TSA Alexa-488 (B40953, Invitrogen).

Stained slides were dehydrated and coverslipped with either Cytoseal 60 (single DAB stains; 8310-4, Thermo Fisher Scientific) or Prolong gold (multiplex stains; P36930, Thermo Fisher Scientific). Positive and negative controls (no primary antibody) were included during staining runs. The slides were digitally scanned at 20× magnification using Aperio AT2 (Aperio Technologies) and uploaded to the Aperio eSlideManager database (Leica Biosystems Inc) at the Pathology Services Core at UNC.

### Interpretation of p16 IHC Histopathology

In addition to the digital image analysis described below, p16 IHC was also previously scored by pathologists for protein expression (79). Each core was scored for cytoplasmic intensity staining and nuclear intensity staining on a 0–3 scale. The percent of positive staining tumor cells was quantified using 10 microscopic fields of 100 cells each. p16 positive was defined as a cytoplasmic or nuclear score of 1–3 in at least 70% cells.

### Digital Imaging and Analysis

DAB-stained slides for SYNGR3 were digitally scanned using the Aperio ScanScope-XT (serial number ss1475, Aperio Technologies). DAB-stained slides for p16 were digitally scanned using Aperio ScanScope CS (serial number ss5072, Leica Biosystems). Multiplex immunofluorescent slides were scanned using the Aperio ScanScope FL (serial number ss6132, Leica Biosystems). All images were scanned at an apparent 20× magnification and uploaded to the Aperio eSlideManager database (version 12.4.3, Leica Biosystems) at the Translational Pathology Laboratory at UNC.

#### Analysis of Single Stained Slides

TMA images stained for p16 or SYNGR3 were digitally segmented into individual cores using TMALab (Aperio Technologies). Whole tissue sections stained were annotated using Aperio ImageScope to remove staining artifacts and tissue folds before they were analyzed. The Cytoplasmic v2 algorithm (Aperio Technologies) was used to analyze p16 and both the Cytoplasmic v2 and the Membrane v9 algorithms (Aperio Technologies) were used to analyze SYNGR3 staining. Using these algorithms, cells were analyzed for DAB signal and the number and percentage of cells with light (1+), medium (2+), and strong (3+) cell staining was determined. H scores were calculated using the following formula: 3 × percentage of strongly staining cells + 2 × percentage of moderately staining cells + the percentage of weakly staining cells, giving a range of 0 to 300. The average DAB intensities in cells, cytoplasm, and nuclei were also determined.

#### Analysis of Multiplex Stained Slides

TMA images were digitally segmented into individual cores using Tissue Studio in IF TMA mode (Tissue Studio version 2.7 with Tissue Studio Library version 4.4.2; Definiens Inc.). Cellular Coexpression analysis algorithms were used to quantify the number of cells expressing individual markers, two of three markers, and all three markers, and cells that were negative for all markers. Tumor microenvironment analysis algorithm was used to segment cores into regions of interest (ROI) based on designated epithelial marker (pan-CK or p16, respectively) and quantify cell number expressing/coexpressing each marker. The average cytoplasmic intensity was also determined for all ROIs. Tumor stroma was defined as 25 μm on either side of the border of the tumor core.

### TMA Data Processing

Cores were binned into categories DN, SP-p16 (SP1), SP-ISH (SP2), and DP as described in Fig. 3 and kept these category designations for all future analyses. For multiplex staining, the number of cells coexpressing SYNGR3, CD3, and pan-cytokeratin was added to the number of cells coexpressing only SYNGR3 and CD3 to get the number of total cells coexpressing SYNGR3 and CD3. Similarly, the number of cells coexpressing SYNGR3, CD45, and p16 was added to the number of cells coexpressing only SYNGR3 and CD45 to get the number of total cells coexpressing SYNGR3 and CD45. Total number of cells expressing SYNGR3 was calculated by adding the number of cells expressing SYNGR3 alone, as well as the number of cells in which SYNGR3 was expressed with other marker(s). The number of cells with a nucleus detected in each core was used as the total cell count to calculate percent of cells expressing a particular marker and/or marker coexpression.

For survival analysis, one core per patient tumor block was selected and cores were binned by p16 or SYNGR3 expression from digital image analysis. High p16 cytoplasmic expression was defined as an H-score of 70 or higher; high nuclear expression as a H-score of 100 or higher. High SYNGR3 expression was defined as a cytoplasmic H-score of 70 or higher. For SYNGR3 expression based on p16 staining groups in the HPV(+) TMA, all DN and HPV(−) by droplet digital PCR (ddPCR) cores were excluded; any SP1 core that also had a DN core in its same patient block were also excluded.

### ROC Curve Analyses

ROC curve analysis was carried out to examine and compare the classification accuracy on HNSC tumor HPV status among several measures, including p16 and SYNGR3 IHC percentage of tumor staining and H-score. For each of the classifications measured, the AUC was calculated using the pROC package in R (80) from which HPV status was determined by ddPCR. The optimal cut-off point was determined by the average sensitivities and specificities.

### Survival Analyses

Summary statistics, univariate and bivariate methods including Kaplan–Meier survival analyses and bivariate tests with a significance level alpha set to <0.05, were used to describe the distribution of our patient sample and present their demographic (age, sex, race, smoking status, alcohol status) and clinicopathologic (tumor site) characteristics overall and by p16 cytoplasmic/nuclear expression status. Crude 5- and 10-year survival rates by p16 status were also calculated.

Multivariable Cox regression models adjusted for age, sex, race, smoking status, alcohol status, and tumor site were created to estimate the associations of p16 status with OS and DSS. To evaluate these associations, we used HRs and corresponding 95% confidence intervals (CI). All statistical analyses were performed with Stata 16.1 (Stata, RRID:SCR_012763, StataCorp LP) and the same program was also used for figure production.

### ddPCR

HPV was detected by ddPCR on the QX-200 platform (Bio-Rad) using QuantaSoft software v1.7.4.0917 (Bio-Rad). The assay was performed as described previously (81, 82); details and primer sequences can be found at Chera and colleagues 2019. The quality of DNA extracted from FFPE tumor blocks was assessed by ddPCR targeting a human genomic locus, estrogen receptor (*ESR1*) gene. Specific primers and hydrolysis probes were designed to amplify a portion of *E6* and *E7* genes encoded by HPV16 while only a portion of *E7* gene was amplified in case of other high risk HPV strains namely, 18, 31, 33, and 35. Two TMA cores which were not included in ddPCR evaluation but were positive by ISH *E6/E7* were included as “true positives” due to the high specificity of HPV ISH. Appropriate HPV plasmid controls were used as positive control for each of the digital PCR assays.

### Statistical Analyses

Statistical analyses were performed with GraphPad Prism (GraphPad Prism version 9, RRID:SCR_002798) using Student *t* test, one-way ANOVA or two-way ANOVA where applicable. Data are presented as mean ± SD or mean ± SEM as indicated in the figure legends.

### Data Availability Statement

The bioinformatics data analyzed in this study were obtained from TCGA and the NCBI GEO at (https://www.ncbi.nlm.nih.gov/geo/). The raw IHC data analyzed for this study were generated at the UNC Pathology Services Core. Derived data supporting the findings of this study are available from the corresponding author upon request.

## Results

### Identification of SYNGR3 within Immune Cells of HPV(+) HNSCs

**FIGURE 1:**
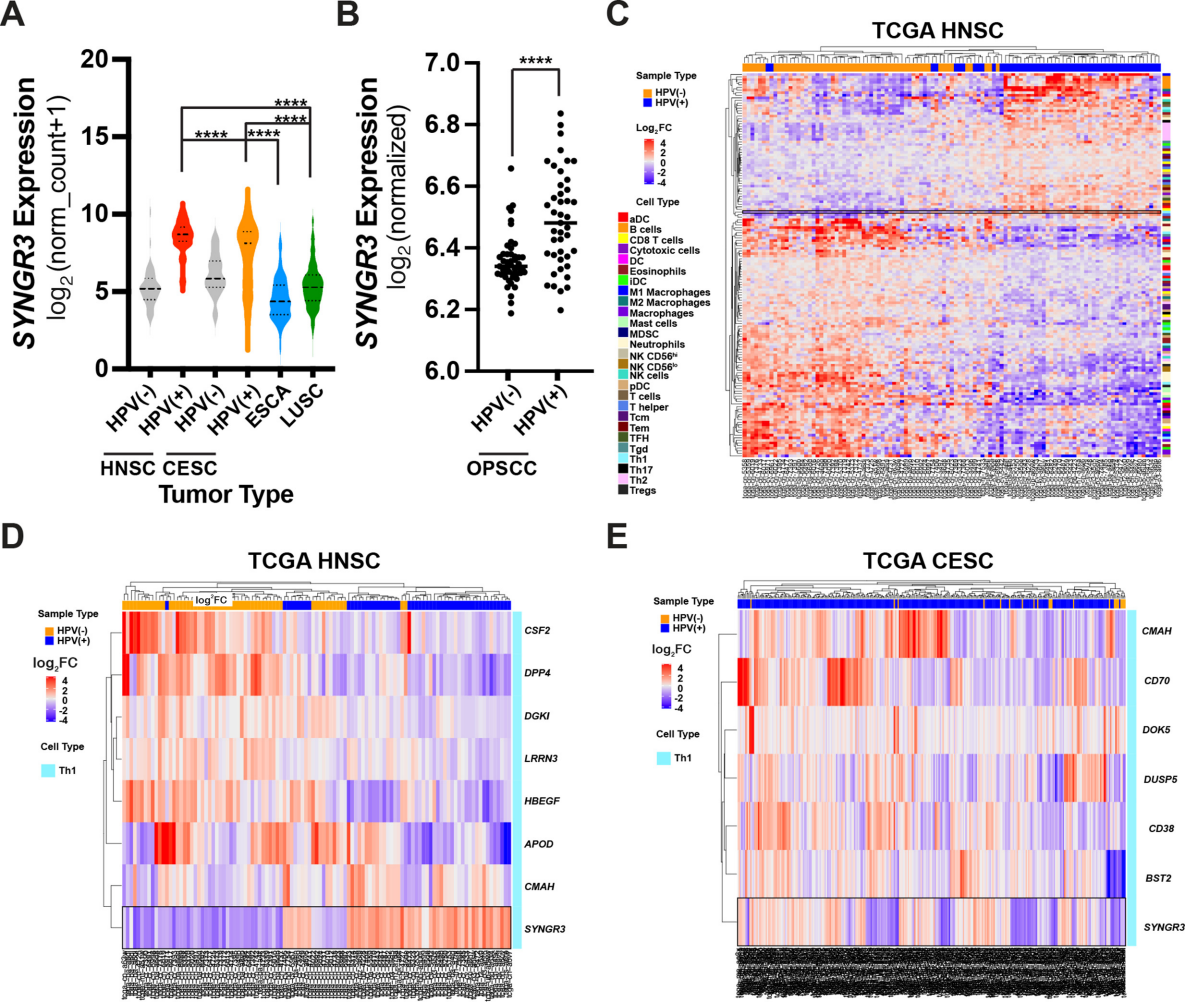
HPV(+) HNSC tumors exhibit a unique immunogenomic signature associated with *SYNGR3*^hi^ Th1 T cells. **A,** Comparison of *SYNGR3* expression according to squamous tumor type and HPV status. Data were extracted from TCGA for HNSC, CESC, ESCA, and LUSC RNA-seq datasets and log_2_ median-centered expression plotted according to HPV status. TCGA = The Cancer Genome Atlas; HPV = human papillomavirus; HNSC = head and neck squamous cell carcinoma; CESC = cervical and endocervical squamous cell carcinoma; ESCA = esophageal squamous cell carcinoma; LUSC = lung squamous cell carcinoma. **B,** Comparison of *SYNGR3* expression according to HPV status. Data for HPV(+)OPSCCs and HPV(−) OPSCCs were extracted from the GSE65858 dataset and log_2_ median-centered expression plotted according to HPV status. Scale of *y*-axis set at the closest integer to the lowest sample values (6.0) to visualize the spread in expression across all of the samples. **C,** Unsupervised hierarchical clustering of immune-related genes (*n* = 1,500) expressed in patients (*n* = 109) from the HNSC TCGA RNA-seq dataset. **D,** Unsupervised hierarchical clustering of immune-related genes (*n* = 8) expressed specifically in Th1 T cells of patients (*n* = 109) from the HNSC TCGA RNA-seq dataset. **E,** Unsupervised hierarchical clustering of immune-related genes (*n* = 8) expressed specifically in Th1 T cells of patients (*n* = 109) from the CESC TCGA RNA-seq dataset.

To identify a biomarker of HPV-positive head and neck cancers, we first determined which genes are differentially expressed between HPV(+) and HPV(−) tumors using TCGA HNSC dataset. Specifically, we examined gene expression in tumors for which clinical information was available. Of the original 279 published HNSC tumors analyzed (9), we categorized samples based on p16 IHC and HPV16 *E6/E7* ISH status which resulted in 53 HPV(+) samples (i.e., positive for both p16 IHC and HPV16 *E6/E7* ISH) and 56 HPV(−) samples (i.e., negative for both p16 IHC and HPV16 *E6/E7* ISH). Unsupervised hierarchical clustering of these data confirmed previous studies (7, 83–85) demonstrating that HPV(+) and HPV(−) tumors have distinct transcriptomic profiles (Supplementary Fig. S1A). *SYNGR3* was fourth most significantly (*P* = 9.40E-79) upregulated gene identified in HPV(+) tumors (Supplementary Table S3 in Supplementary Materials and Methods S1), an observation previously identified using an independent patient cohort (7), but to date *SYNGR3* has yet to be further investigated. Notably, analysis of CESCs, which are known to be predominantly driven by HPV, revealed that *SYNGR3* is also differentially expressed in these tumors based on HPV status (Supplementary Fig. S1B and Supplementary Table S4 in Supplementary Materials and Methods S1). To further test the association between *SYNGR3* expression and HPV status, we next analyzed all available TCGA PanCancer squamous cell carcinoma datasets and confirmed that expression of *SYNGR3* is significantly elevated only in HPV(+) tumors (Fig. 1A). Moreover, additional analyses confirm that *SYNGR3* expression is high in HPV(+)OPSCC and low in HPV(−) OPSCC (Fig. 1B), suggesting that SYNGR3 is indeed a diagnostic and prognostic biomarker of head and neck cancers.

Despite recent progress that has identified a role for SYNGR3 in regulating synaptic vesicles and neuronal function (70, 86–88), a significant knowledge gap remains in our understanding of SYNGR3 biology. However, two closely related family members, SYNGR1 and SYNGR2, were shown to be expressed within immune cells (89, 90). Given this potential neuronal-independent role for SYNGR3 in immunobiology and the unique tumor-immune landscape that exists between HPV(+) and HPV(−) HNSCs (68), we next examined immune-related gene expression profiles. We applied our “Modified Immunome Signature” based on curated immunogenomic signatures (60, 62, 73) and performed unsupervised hierarchical clustering of differentially expressed genes (DEGs) in HPV(+) HNSC and (HPV+) CESC (Fig. 1C and Supplementary Fig. S1C in Supplementary Materials and Methods S1). We then clustered these genes according to all 26 immune cell subtypes as defined by Porrello and colleagues (73). Notably, the Th1 T-cell subtype displayed a significant correlation with *SYNGR3* expression in both HPV(+) HNSC (log_2_FC = 3.26, SE = 0.17, *P* = 9.4E-79, *q*-value = 4.55E-75) and HPV(+) CESC (log_2_FC = 1.60, SE = 0.31, *P* = 2.75E-07, *q*-value = 6.42E-06) compared with HPV(−) tumors (Fig. 1D–E; Supplementary Table S3 and S4 in Supplementary Materials and Methods S1). These findings suggested that SYNGR3 may be a useful tumor cell extrinsic biomarker for defining HPV status.

### Validation of SYNGR3 Expression in HPV(+) HNSC Cohorts

**FIGURE 2:**
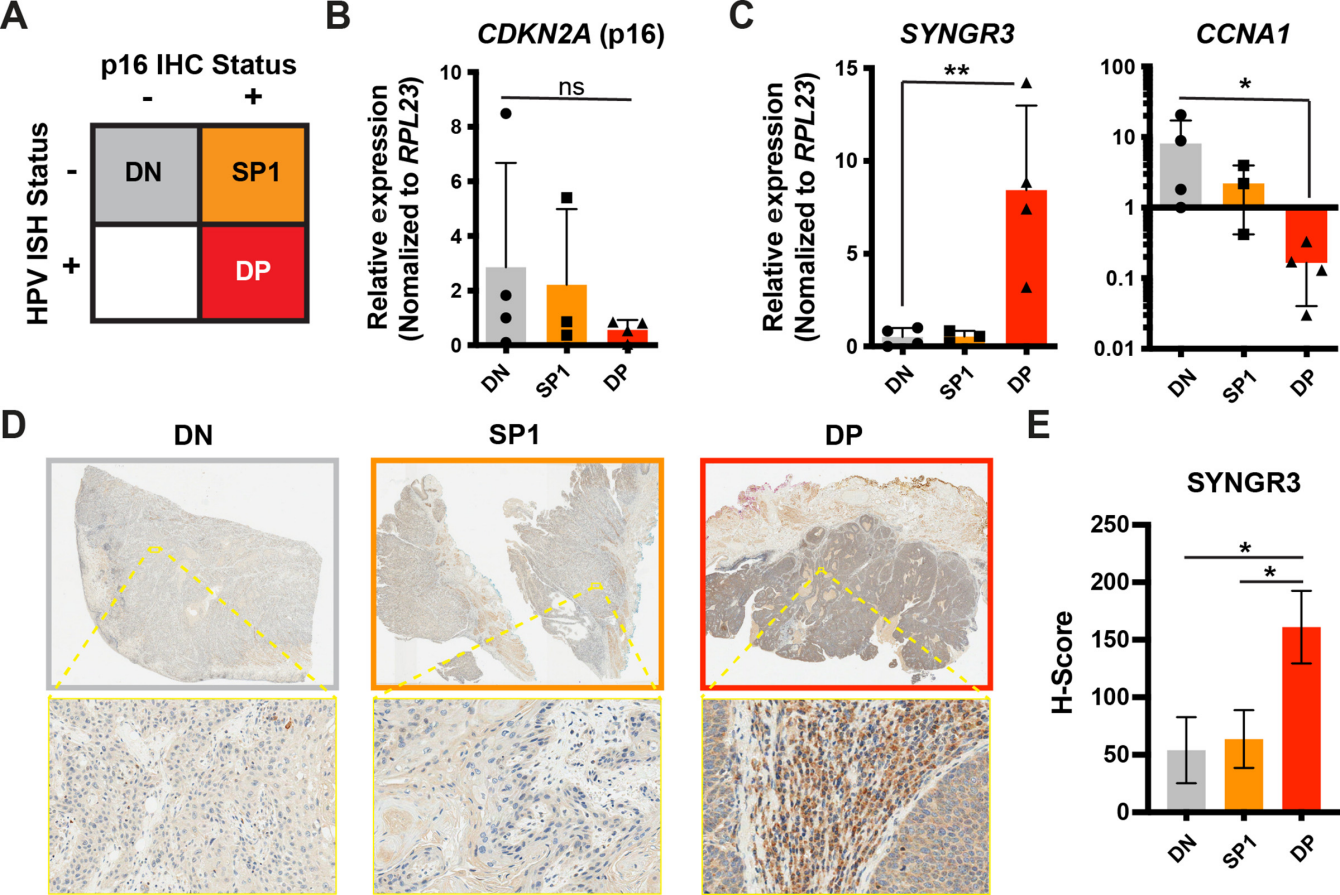
Validation of elevated SYNGR3 mRNA and protein in HPV(+) HNSC patient tumors. **A,** Schematic of fresh-frozen human HNSC patient tumors categorized by HPV assay clinical diagnoses, including p16 IHC and HPV16 ISH. DN = double negative (*n* = 4), SP1 = single positive for p16 IHC (*n* = 3), DP = double positive for p16 IHC and HPV16 ISH (*n* = 3). **B,** qRT-PCR analysis of *CDKN2A* (gene name for p16 protein) mRNA levels. *CDKN2A* expression was normalized to *RPL23* mRNA levels and fold expression was calculated relative to the average of the DN group. Data are presented as the mean ± SEM (*n* = 4 technical replicates; one-way ANOVA test, ns = not significant). **C,** qRT-PCR analysis of *SYNGR3* and *CCNA1* mRNA levels. *SYNGR3* and *CCNA1* expression were normalized to *RPL23* mRNA levels and fold expression was calculated relative to the average of the DN group. Data are presented as the mean ± SEM (*n* = 4 technical replicates; one-way ANOVA test; *, *P* < 0.05; **, *P* < 0.01). **D,** Analysis of SYNGR3 protein expression in the fresh-frozen tumor validation cohort. Representative 1× and high magnification 20× inset images of SYNGR3 IHC staining within the tumor and stroma of sections according to each respective HPV assay category. **E,** Quantification of SYNGR3 IHC staining in **D** represented as H-score. Data are presented as mean ± SEM (*, *P* < 0.05).

To begin validating the clinical relevance of increased SYNGR3 expression, we first obtained a cohort of 11 human primary HNSC samples with available clinical information regarding HPV status from the surgical pathology department at the University of North Carolina (UNC) Hospitals (Chapel Hill, NC). We performed qPCR on fresh-frozen specimens to determine levels of the canonical HPV biomarker p16, as well as other candidate genes identified in Fig. 1 as being differentially expressed specifically in HPV(+) HNSCs (Supplementary Fig. S2A and S2B in Supplementary Materials and Methods S1). The tumor specimens were separated into three distinct categories based on clinical results from two HPV assays (Fig. 2A): single positive 1 (SP1, positive for p16 IHC; *n* = 3), double positive (DP, positive for both p16 IHC and HPV ISH; *n* = 3), and DN (double negative, *n* = 4). There were no available samples positive for only HPV ISH in this tumor cohort. While mRNA levels of *CDKN2A*/p16 unexpectedly did not correlate with HPV status (Fig. 2B), we observed a significant upregulation of *SYNGR3* mRNA (*P* < 0.01) in DP tumors (Fig. 2C). Alternatively, *CCNA1* was identified in our bioinformatic analyses as being inversely correlated to HPV status and qPCR confirmed that its expression is significantly higher (*P* < 0.05) in the DN group compared with the DP group (Fig. 2C). To validate these findings at the protein level, we next performed IHC on FFPE sections of these tumors. Concordant with the results obtained for mRNA expression, we found that staining for SYNGR3 protein was significantly higher (*P* < 0.05) in DP samples than in SP1 or DN samples (Fig. 2D and E). Thus, although p16-positive tumors in the CHANCE cohort are often negative for HPV E6/E7, we observed that high levels of SYNGR3 are only associated with tumors positive for HPV assays with high specificity (i.e., SP2 and DP tumors).

**FIGURE 3:**
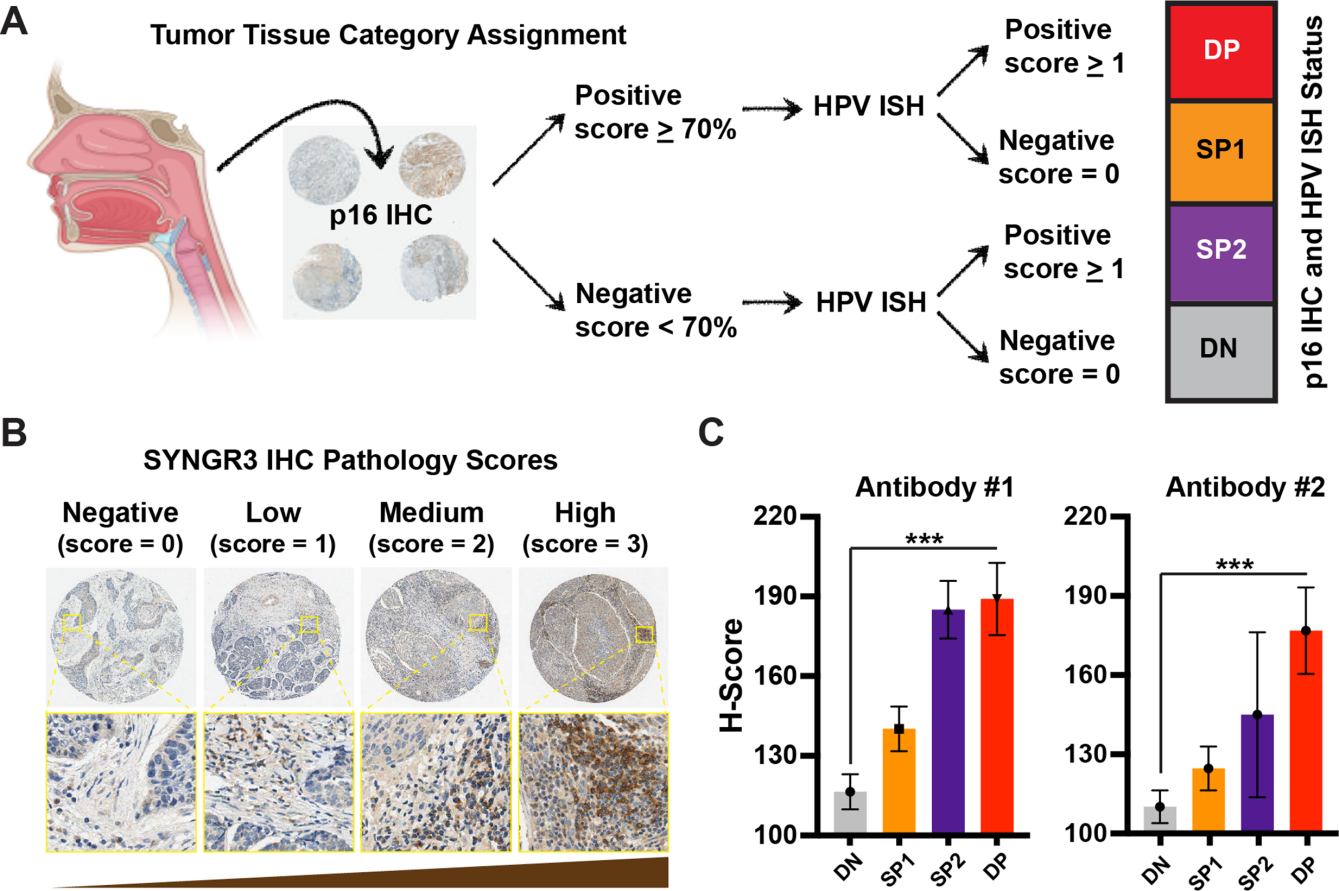
Antibody validation confirms that SYNGR3 protein is expressed at significantly higher levels in HPV(+) HNSC. **A,** Schematic of TMA composed of FFPE HNSC patient tumor cores categorized by HPV assay clinical diagnoses, including p16 IHC and HPV16 ISH. DN = double negative (*n* = 103), SP1 = single positive for p16 IHC (*n* = 69), SP2 = single positive for HPV16 ISH (*n* = 5), DP = double positive for p16 IHC and HPV16 ISH (*n* = 13). Positive p16 IHC cores were defined as equal to or greater than 70% of cells with a score of 1, 2, or 3 in either the nucleus or cytoplasm. HPV ISH positive cores were defined as any cells with a nuclear score of 1, 2, or 3. **B,** Comparison of SYNGR3 protein expression in HNSC patient tumors. Representative images and high magnification 20× inset ROIs of SYNGR3 IHC staining depicting SYNGR3 expression within the tumor stroma shown for cores with the highest percentage of stained cells in each category of IHC scores [0 = negative (no staining for SYNGR3); 1 = low; 2 = medium; 3 = high]. **C,** Quantification of SYNGR3 IHC staining of CHANCE TMA by HPV assay category delineated in **A** represented as H-score. Data are presented as mean ± SEM (***, *P* < 0.001).

Given the sample size limitations of our initial validation cohort, we sought to extend these findings to a larger panel of HNSC tumors. Thus, we next performed SYNGR3 IHC on a TMA that we previously generated for which the results of HPV clinical assays (p16 IHC and HPV ISH) were available (78, 79, 91). These HNSC tumor tissues were collected from patients across North Carolina as part of our CHANCE study and assigned to categories (e.g., DP, SP1, SP2, or DN) according to the clinical assay results (Fig. 3A; Supplementary Table S5 in Supplementary Materials and Methods S1). Results from both assays were graded by two independent pathologists, as published previously (79). The cutoff for p16 positivity was defined as an IHC signal intensity of 1, 2, or 3 in at least 70% of the tumor, and the cutoff for HPV ISH positivity was defined as an ISH signal intensity of 1, 2, or 3 in cell nuclei (Fig. 3B). Using these criteria for HPV marker positivity, we found a similar relationship between increased SYNGR3 expression and HPV marker positivity in this statewide panel of CHANCE tumors using two independent antibodies purchased from different vendors (Fig. 3C) similar to that observed with the UNC Hospitals cohort (Fig. 2D and E). Notably, despite being the more sensitive assay, p16 IHC is prone to generating false negatives due to suboptimal specificity for HPV infection. Therefore, the increased sample size offered by the TMA allowed for inclusion of a fourth category of samples with high specificity, SP2, which are positive for HPV ISH but negative for p16 IHC. Collectively, these data suggest that elevated SYNGR3 has high specificity for HPV(+) HNSC cases and further support its use as a novel tumor cell extrinsic biomarker of HPV infection.

### Codetection of SYNGR3 IHC and p16 IHC Enhances Specificity for HPV(+) HNSC

We next evaluated the performance of p16 IHC and SYNGR3 IHC in comparison with a “gold standard” assay for HPV16 DNA that we recently validated in our ongoing multi-institutional prospective phase II clinical trials for patients with HPV-associated HNSC (81, 82, 92, 93). Specifically, the true HPV status was determined on a subset of the CHANCE specimens identified from the TMA by our highly sensitive and specific ddPCR assay for viral genes of high-risk HPV (Supplementary Table S2 in Supplementary Materials and Methods S1). The SYNGR3 IHC assay had very high specificity (89.7%) and positive predictive values (PPV, 82.4%) for HPV16 E7 DNA compared with p16 IHC specificity (72.4%) and PPV (75.0%) in the TMA cohort (Table 1). Moreover, these results highlight the lack of specificity of p16 IHC for HPV detection, as this subset included 11 false positives (Table 1).

**TABLE 1:** p16 IHC and SYNGR3 IHC sensitivity and specificity analyses

	Gold standard HPV test results^a^								
Test	Negative	Positive	Sensitivity (95% CI)	Specificity (95% CI)	PPV	NPV	AUC	*P*	FDR
p16 IHC (*n* = 58)									
H-score			85.7% (57.1–96.4)	72.4% (6.9–82.8)	75.0%	84.0%	0.835 (0.701–0.924)	1.04E-05	8.35E-05
% Positive cells			78.6% (46.4–92.9)	72.4% (27.6–86.2)	73.3%	77.8%	0.784 (0.663–0.899)	1.45E-04	2.91E-04
Negative	18	6							
Positive	11	23							
SYNGR3 IHC (*n* = 58)									
H-score			50.0% (17.9–64.3)	89.7% (55.2–100.0)	82.4%	65.0%	0.729 (0.589–0.851)	3.06E-03	3.77E-03
% Positive cells			50.0% (0.0–67.9)	89.7% (55.2–100.0)	82.4%	65.0%	0.717 (0.575–0.839)	5.09E-03	5.81E-03

^a^The gold standard test for HPV status is defined by detection of HR-HPV (strains 16, 18, 31, 33, and 35) by ddPCR assay (*see* Chera BS, et al. 2019).

**FIGURE 4:**
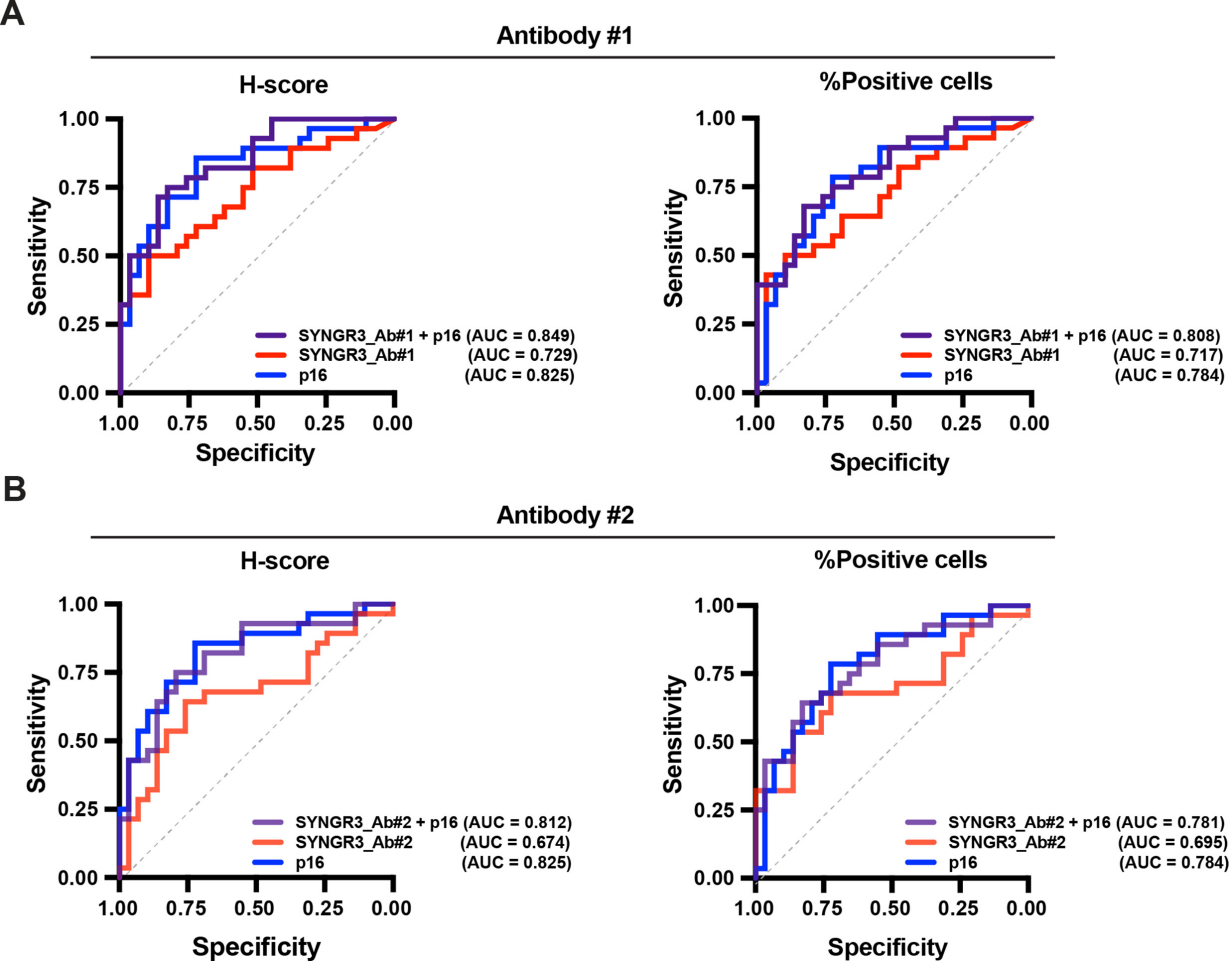
Codetection of SYNGR3 and p16 provides a tractable pair of IHC-only biomarkers for identifying HPV status in HNSC. ROC curves plotting sensitivity by specificity of H-score (left) and percent positive cells (right, cells scoring 1–3) using two independent antibodies (**A** and **B**) for SYNGR3 IHC staining of CHANCE TMA cases with known HPV status as determined by ddPCR. AUC = area under the curve.

We performed ROC analyses to determine the optimal cut-off points for the two different antibodies examined in this study for SYNGR3 IHC interpretation in comparison with p16 IHC (Fig. 4A and B; Table 1). The AUC for H-score (AUC = 0.729) was higher for Antibody #1 than that for percentage positive stained cells (AUC = 0.717), while the AUC for percentage positive stained cells (AUC = 0.695) for was higher for Antibody #2 than that for H-score (AUC = 0.674). Regardless of the single classification method used, the combination of SYNGR3 IHC and p16 IHC was better at discriminating true tumor HPV status with SYNGR3 Antibody #1 (Fig. 4A). An optimal H-score cut-off point of 71.64 on a scale of 0 to 300 for SYNGR3 IHC yielded an average sensitivity of 50.0% and specificity of 89.7% for HPV detection. Thus, when used in combination, the superior specificity of SYNGR3 IHC elevates the diagnostic capabilities of p16 IHC in determining true HPV status, suggesting that comprehensive characterization of the cells expressing SYNGR3 and their location within HPV(+) HNSCs will benefit its clinical application.

### SYNGR3 Expression is Confined to T and B Cells within the Tumor Stroma

**FIGURE 5:**
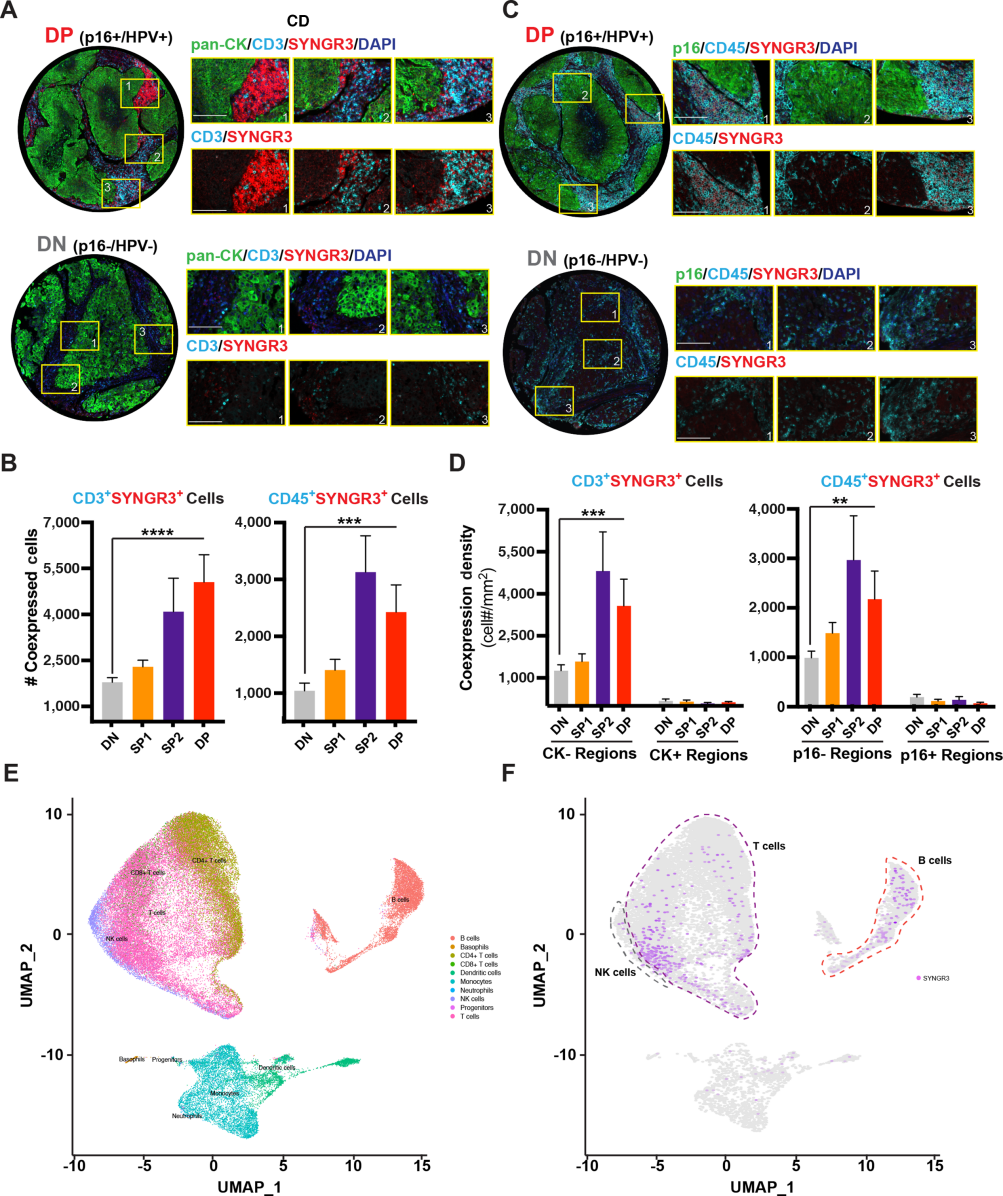
T and B cells in the stromal compartment have the strongest correlation with high SYNGR3 expression. **A,** Representative multiplex IHC staining of TMA cores for SYNGR3 (red), CD3 (cyan, pan T-cell marker), pan-CK (green, pan-cytokeratin), and DAPI (purple, nuclei) with enumeration of SYNGR3^+^/CD3^+^ cells in CK+ and CK− regions. Scale bar = 400 μm. **B,** Quantification of IHC of SYNGR3 and CD3 (T cells; left) or SYNGR3 and CD45 (all immune cells; right) according to HPV assay category. Data represented as number of coexpressing cells in all ROIs. Expression data are presented as mean ± SEM (***, *P* < 0.001; ****, *P* < 0.0001). **C,** Representative multiplex IHC staining of TMA cores for SYNGR3 (red), CD45 (cyan, pan immune cell marker), p16 (green, tumor), and DAPI (purple, nuclei) with enumeration of SYNGR3^+^/CD3^+^ cells in p16+ and p16− regions. Scale bar = 400 μm. **D,** Quantification of IHC of SYNGR3 and CD3 (T cells; left) or SYNGR3 and CD45 (all immune cells; right) according to HPV assay category separated by epithelial/stromal ROIs. Epithelial/tumor regions were defined by either pan-CK (left) or p16 IHC (right), and included the tumor stroma analysis (defined by 25 μm on either side of tumor border). Data represented as density of coexpressing cells. Expression data are presented as mean ± SEM (**, *P* < 0.01; ***, *P* < 0.001). **E** and **F,** Single-cell RNA-seq data of HNSC HPV(+) tumors confirms *SYNGR3* expression in T and B cells. Expression data are presented as uniform manifold approximation and projection (UMAP) plots.

Our immunogenomic analyses and initial identification of elevated *SYNGR3* expression in HPV(+) HNSCs indicated that it is highly expressed within the Th1 subset of tumor-infiltrating T cells (Fig. 1C). To examine this more closely and characterize the number and location of SYNGR3^+^ cells, we performed multiplex IHC to examine SYNGR3 expression in T cells and more generally hematopoietic cells, as well as their distribution within different tumor compartments. We first stained for SYNGR3, CD3 (pan T-cell marker), and CK (pan-cytokeratin marker to define the epithelial compartment) and found that the number of coexpressed SYNGR3^+^/ CD3^+^ cells was significantly higher (∼3-fold, *P* < 0.0001) in DP (p16 IHC+/HPV ISH+) tumors compared with DN (p16 IHC-/HPV ISH−) tumors (Fig. 5A). The average number (and percentage) of cells coexpressing SYNGR3^+^/ CD3^+^ in each category are as follows: DN = 1,787 cells (20.4% of total), SP1 = 2,291 cells (23.4%), SP2 = 4,088 cells (47.6%), and DP = 5,054 cells (43.3%; Fig. 5B; Supplementary Fig. S3A in Supplementary Materials and Methods S1). This difference was validated within the samples with confirmed HPV status by ddPCR (Supplementary Fig. S3B in Supplementary Materials and Methods 1). Interestingly, only approximately 50% of all SYNGR3^+^ cells were identified as being dual-positive SYNGR3^+^CD3^+^ cells (Supplementary Fig. S3C in Supplementary Materials and Methods S1). To validate the immunogenomic analyses, multiplex IHC with additional T-cell markers revealed that SYNGR3 expression colocalizes with CD4^+^ T cells but not with CD8^+^ cytotoxic T cells within the tumor stroma (Supplementary Fig. S3D in Supplementary Materials and Methods S1). Collectively, these findings suggest that additional immune cell types may also express SYNGR3.

To examine this further, we next stained for SYNGR3, CD45 (pan-hematopoietic cell marker), and p16, and found that the number of coexpressed SYNGR3^+^/ CD45^+^ cells was significantly higher (∼2.5-fold, *P* < 0.001) in DP (p16 IHC+/HPV ISH+) tumors compared with DN (p16 IHC−/HPV ISH−) tumors (Fig. 5B and C). Similarly, the average number of cells coexpressing SYNGR3^+^/ CD45^+^ in each category was: DN = 1,042 cells, SP1 = 1,405 cells, SP2 = 3,129 cells, and DP = 2,425 cells. ROI analyses of SYNGR3^+^ cell compartmentalization within the tumor microenvironment were performed by defining the tumor as 25 μm on either side of the border of the tumor core and revealed that SYNGR3^+^/CD3^+^ and SYNGR3^+^/CD45^+^ cells were primarily confined to stromal regions (pan-CK and p16 negative) compared with the cancer cell islet (pan-CK and p16 positive) regions (Fig. 5D; Supplementary Fig. S3E in Supplementary Materials and Methods S1). To complement the multiplex IHC analyses and reveal the identity of these SYNGR3^+^ immune cells, we analyzed previously published single-cell RNA-seq data generated from purified CD45^+^ cells (i.e., all immune cells) for *SYNGR3* expression in HPV(+) HNSCs (68). Unsupervised clustering of these immune cells confirmed *SYNGR3* expression within T cells but also within B cells of HPV(+) HNSCs (Fig. 5E and F). Notably, B cells and the presence of tertiary lymphoid structures (TLS) can predict immune checkpoint inhibitor efficacy and influence outcome in patients with HNSC, suggesting that detection of SYNGR3 may also have prognostic value (57).

### SYNGR3 Expression is Associated with Improved Survival

**FIGURE 6:**
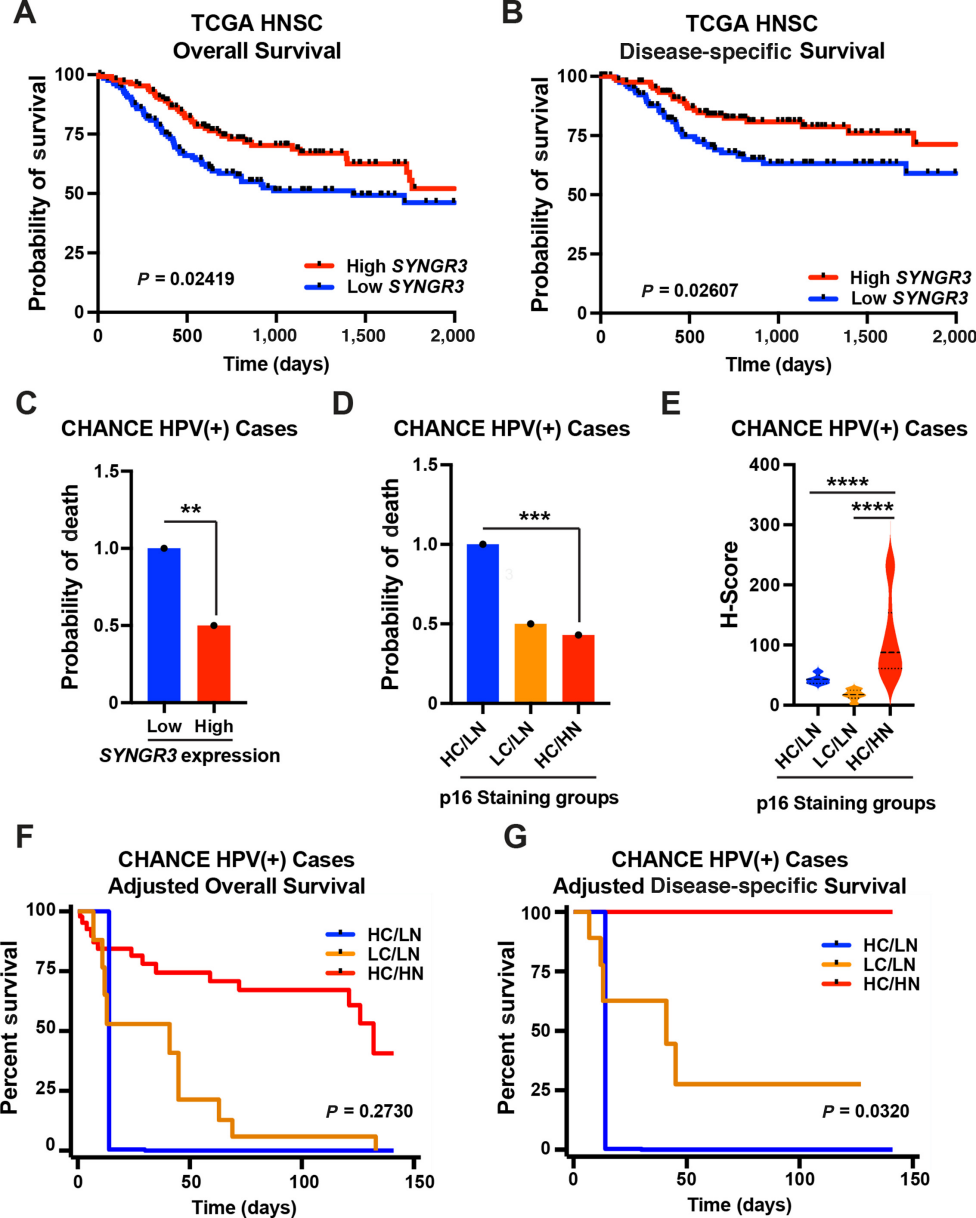
Relationship between high SYNGR3 expression and DSS of patients with HPV(+) HNSC. Kaplan–Meier curves for OS (**A**) and DSS (**B**) of patients with TCGA HNSC stratified by *SYNGR3* expression from tumor RNA-seq data using UCSC Xenabrowser. High *SYNGR3* = top quartile mRNA expression, Low *SYNGR3* = bottom quartile mRNA expression; *P* < 0.05. **C,** Probability of death in HPV(+) CHANCE TMA patients stratified according to SYNGR3 expression levels. (**, *P* < 0.01). **D,** Probability of death in HPV(+) CHANCE TMA patients stratified by p16 cytoplasmic and nuclear expression by p16 IHC into localization categories. High cytoplasmic (HC) = cytoplasmic H-score of 50 and above; low cytoplasmic (LC) = cytoplasmic H score below 50; high nuclear (HN) = nuclear H-score of 70 and above; low nuclear (LN) = nuclear H-score below 70. (***, *P* < 0.001). **E,** Comparison of SYNGR3 protein expression by IHC stain of whole HPV(+) CHANCE TMA cores separated by the p16 localization categories defined in **D**. Expression data are presented as violin plot of H-score and presented as mean ± SEM (****, *P* < 0.0001). **F** and **G,** Survival curves of following multivariate analysis of HPV(+) CHANCE TMA patients adjusted for age, sex, race, smoking status, alcohol status, and tumor site. Patients separated by p16 localization category defined in **D**. Unadjusted curves can be found in Supplementary Fig. S4.

To evaluate the utility of SYNGR3 as a novel prognostic immune cell biomarker of HPV(+) HNSC, we used the UCSC Xenabrowser to analyze publicly available RNA-seq data for TCGA head and neck cancer dataset (94). Stratifying samples into *SYNGR3* expression quartiles revealed a marked separation in the survival curves such that patients with high *SYNGR3* expression show significantly better OS (*P* = 0.0242) and DSS (*P* = 0.02607) compared with patients with low *SYNGR3* expression (Fig. 6A and B; Supplementary Fig. S4A in Supplementary Materials and Methods S1). Using our ROC curve analyses to define the optimal cutoff of SYNGR3 positivity (cytoplasmic H-score of 70), we confirmed that patients included in our TMA with high SYNGR3 expression also have better disease specific survival compared with patients with low SYNGR3 expression (Fig. 6C).

Previous studies demonstrated that p16 cellular localization is an important prognostic biomarker in HNSC (79, 95). We next applied our cytoplasmic and nuclear p16 H-scores to categorize the HPV(+) HNSC TMA into the following groups: high cytoplasmic, high nuclear (HC/HN, *n* = 25); low cytoplasmic, low nuclear (LC/LN, *n* = 13); and high cytoplasmic, low nuclear (HC/LN, *n* = 6; Supplementary Table S5 in Supplementary Materials and Methods S1). This organization of samples reflected those published previously (79), in which the HC/LN group displayed worst DSS (Fig. 6D), with a HR of 8.6 compared with the HC/HN group (*P* = 0.032; Table 2). Remarkably, SYNGR3 expression is significantly higher in the HN/HC group compared with the HC/LN (*P* < 0.001) and LC/LN (*P* < 0.0001) groups and multivariate analysis demonstrates that this group is associated with significantly improved DSS (Fig. 6E and G; Supplementary Fig. S4B and S4C and Supplementary Table S6 in Supplementary Materials and Methods S1). Therefore, SYNGR3 is expressed more highly in the group with improved DSS (HN/HC), suggesting value as a prognostic biomarker in patients with HNSC. Collectively, these findings support a diagnostic and prognostic role for SYNGR3 in HNSC, and potentially other HPV(+) cancers, and indicate that detection of SYNGR3 can be accomplished by pathologic analysis of the tumor stroma, making it distinct yet complementary to currently available HPV detection assays.

**TABLE 2:** Association of demographic, clinicopathologic variables, and p16 cytoplasmic/nuclear status with Cox regression modeling in patients with HNSC from the CHANCE study^a^

	OS			DSS		
Characteristic	HR	95% CI	*P*	HR	95% CI	*P*
**Sex**						
Female	*Ref.*			*Ref.*		
Male	0.36	0.10–1.33	0.124	0.21	0.02–1.76	0.149
**Race**						
Black/African American	*Ref.*			*Ref.*		
White	1.13	0.39–3.34	0.819	2.83	0.35–23.01	0.331
**Alcohol status**						
Heavy use:	*Ref.*			*Ref.*		
>6 months ago						
Heavy use currently:	1.11	0.28–4.45	0.886	11.12	1.33–92.85	0.026
>3 drinks per day						
Light or moderate	0.64	0.18–2.22	0.48	1.98	0.23–17.40	0.537
Never drinker	0.2	0.04–0.92	0.039	0.19	0.02–1.64	0.129
Unknown	.			.		
**Smoking Status**						
Never smoker	*Ref.*			*Ref.*		
Current smoker	0.62	0.13–3.10	0.562	1.96	0.12–30.67	0.633
Unknown	.			.		
**Tumor site**						
Hypopharynx	*Ref.*			*Ref.*		
Larynx	0.93	0.20–4.28	0.925	5.41	0.32–92.27	0.243
Oral	0.4	0.11–1.46	0.166	4.4	0.33–58.41	0.261
Oropharynx	0.42	0.09–1.86	0.252	3.41	0.25–46.38	0.357
**p16 status**						
HC/HN	*Ref.*			*Ref.*		
HC/LN	2.15	0.55–8.48	0.273	8.6	1.21–61.25	**0.032**
LC/LN	1.25	0.42–3.72	0.687	2.87	0.45–18.11	0.263

^a^p16 cytoplasmic/nuclear status HRs are adjusted for sex, race, smoking, alcohol intake, and tumor site.

## Discussion

Broadly, biomarkers can be used to diagnose disease, classify disease subtype, measure response to treatment, and/or monitor disease outcomes. Biomarkers vary by type (e.g., molecular, histologic, radiographic, digital, or physiologic), source (e.g., saliva, tumor biopsy, etc.), and measurement method (96, 97). In the context of cancer, biomarker use includes estimating the risk of developing cancer, routine screening, differential diagnoses, determining disease prognosis, predicting response to therapy, monitoring disease recurrence, measuring drug responses, or monitoring metastatic progression and recurrence (98). Biomarkers can be identified using biology of the tumor as a guide, or by using a discovery-based approach as presented in this study, both of which require extensive clinical validation before application to patient care. This includes establishing analytic validity of the biomarker (99, 100), such as determining its sensitivity and specificity, as well as the clinical validity and utility (101, 102), which have specific guidelines for evaluation and reporting (100, 103–106). Currently, there are various radiographic biomarkers for HNSC patient outcome, namely using PET-CT scans rather than clinical evaluation and CT alone (107–110) and tumor volume (110–121). However, knowledge of HPV status alone is also a prognostic biomarker for HNSC in its own right, indicating better survival compared with HPV(−) (122) Unfortunately, a subset of HPV(+) individuals do not display improved survival (11, 13, 123–128), and therefore, a need exists for criterion to further stratify this subset of HPV(+) patients for guiding treatment decisions.

HPV(+) tumors have increased tumor immune infiltrates, which have also been shown to be positive for prognosis (57, 68, 129–131). With the introduction of immunotherapies in recent years, there has been a focus on identifying biomarkers for response to such treatments. The presence of TLS is an indicator of immunotherapy response and outcome (132–134). Notably, robust Th1 cell infiltration, the cell type we first identified as having increased *SYNGR3* expression in HPV(+) HNSC and CESC, is also associated with improved response to immunotherapy therapy (133). Interestingly, recent studies have also shown the prognostic value for B cell infiltrates in patient outcomes (57) as well as a role for Th1 cells in promoting B-cell function and activity (135), suggesting an intriguing and important role for SYNGR3-expressing B cells in modifying the tumor immune microenvironment. Although Ruffin and colleagues does not identify *SYNGR3* as being differentially expressed in B cells, it does identify the related synaptogyrin family member, *SYNGR2*, as a DEG in its B-cell genetic signature (57). Probing for SYNGR3 in our immunocompetent HPV mouse model (77), as well as in a larger, prospective study could establish whether *SYNGR3* levels are predictive of treatment response, especially for immune-based therapies.

SYNGR3 has primarily been described as a neuronal synaptic gene (69, 70, 86–88); however, some studies have documented altered SYNGR3 expression in the context of cancer (7, 136) although the significance of these observations has not been further investigated. For example, SYNGR3 was included in a prognostic gene signature as being downregulated in chemotherapy-resistant breast cancer cell lines where addition of a histone deacetylase inhibitor (SAHA) was effective (137), supporting our finding that *high* SYNGR3 may indicate better prognosis. SYNGR3 was also identified as being downregulated in a subgroup of HNSCC with high beta-adrenergic signaling, which was also associated with HPV negativity (138), and a known indicator of worse prognosis (139). Finally, SYNGR3 is differentially expressed in chromophobe renal cell carcinoma compared with nonmalignant oncocytomas and used in a 14-gene probe to distinguish between the two with high degree of accuracy (140).

Given the role of SYNGR3 in synaptic vesicle signaling, it would be interesting to evaluate whether SYNGR3 is somehow involved in mediating immune cell signaling in HNSCC. This is especially intriguing given recent studies which identified differential protein cargo within extracellular vesicles between HPV(+) and HPV(−) tumors (141, 142). SYNGR3 directly interacts with vesicle and transport proteins (e.g., TTPA, ARFIP1, and SH3GLB1), proteins with metabolic and growth function (e.g., ACSF2, NRDG4, and PNKP), as well as proteins with known immune function such as SPG21 which may be involved in regulating T-cell function, or MPP1 which regulates neutrophil polarity (143). Future studies aimed at investigating the role of SYNGR3 in HPV biology and immune response in HNSCCs is merited.

Several prognostic biomarkers of HNSC have been identified over the past decade including expression of the estrogen receptor α (ERα) and TRAF3/CYLD (144, 145). While the diagnostic potential of SYNGR3 was thoroughly evaluated herein across several very large and independent cohorts, some limitations in the current study remain. Notably, the diagnostic value of the CHANCE cohort is limited by the number of HPV(−) OPSCC tumors and the prognostic value in the current study is limited by a relatively small number of patients who are primarily smokers. This latter limitation is particularly notable when broken down to the HPV(+) patient subset in our survival analyses. However, the prognostic effect of SYNGR3 expression remained significant after controlling for multiple factors including smoking. It is important to note that we were unable to perform ddPCR on all the tumor blocks due to biospecimen availability, which likely negatively influenced our ROC curve analyses; and increasing the number of samples is predicted to strengthen the sensitivity and specificity measures. We note that p16 is a prognostic marker in its own right, regardless of HPV status (26, 39, 79, 146–152), and our survival analyses did not correct for that. Therefore, these findings could be strengthened by expanding to a larger and more inclusive HNSCC patient cohort. Finally, we lack an actionable clinical grade SYNGR3 antibody, so improving upon these resources would allow for more precise analyses.

Despite HPV infection being a well-established indicator of improved HNSCC outcomes, the reliability of currently available diagnostic assays can be improved. Similarly, increased interest in the tumor immune microenvironment in recent years has revealed its importance in evaluating patient prognosis, as well. This, combined with recent studies detailing disparate genetic signatures and tumor-immune landscapes depending on HPV infection status, motivated our immunogenomic approach to identifying an improved biomarker for HPV diagnosis. Further evaluation of SYNGR3 biology and clinical validation as a biomarker in HNSCC could directly influence patient care.

## Supplementary Material

Figure S1 HPV(+) and HPV(-) HNSC are characterized by distinct immunogenomic signatures.

Figure S2 Validation of upregulated and downregulated genes in HPV(+) HNSC patient tumors.

Figure S3 Additional multiplex staining coexpression and ROI quantification.

Figure S4 Association of SYNGR3 expression patient survival.

Supplementary Table ST1 List of Primers.

Supplementary Table ST2 Demographic and clinical characteristics of the study cases.

Supplementary Table ST3 Differentially expressed genes from the TCGA Head and Neck Squamous Cell Carcinoma (HNSC).

Supplementary Table ST4 Differentially expressed genes from the TCGA Cervical Squamous Cell Carcinoma (CESC).

Supplementary Table ST5 Descriptive statistics for HNSC patients from the CHANCE study by p16 cytoplasmic/nuclear status.

Supplementary Table ST6 Crude 5- and 10-year survival rates by p16 cytoplasmic/nuclear status.
