# Supplementary material for: Tumor Cell Extrinsic Synaptogyrin 3 Expression as a Diagnostic and Prognostic Biomarker in Head and Neck Cancer
Source: Cancer Res Commun. 2022 Sep 15;2(9):987–1004. doi: 10.1158/2767-9764.CRC-21-0135 (PMC9491693; doi:10.1158/2767-9764.CRC-21-0135)
Supplement: Figure S1 — HPV(+) and HPV(-) HNSC are characterized by distinct immunogenomic signatures. [file crc-21-0135-s01.docx]

**
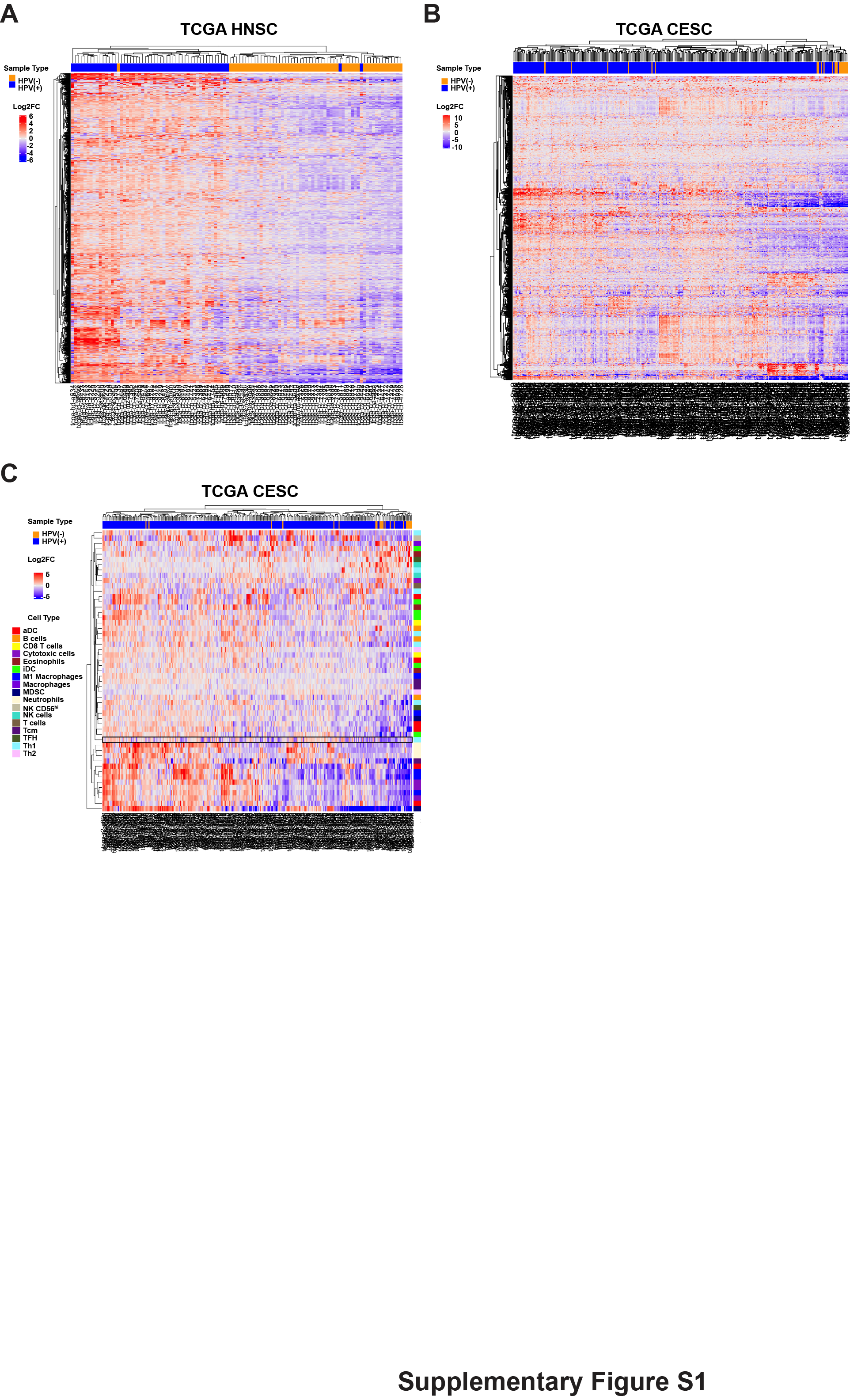
**

**Supplementary Figure S1. HPV(+) and HPV(-) HNSC are characterized by distinct immunogenomic signatures.**

1. Unsupervised hierarchical clustering of all upregulated genes (n = 1079) expressed in all cell types of patients (n = 109) from the HNSC TCGA RNA-seq dataset.
2. Unsupervised hierarchical clustering of genes (n = 1175) expressed in all cell types of patients (n = 303) from the CESC TCGA RNA-seq dataset.
3. Unsupervised hierarchical clustering of immune-related genes (n = 1500) expressed across 26 immune cell types of patients (n = 109) from the CESC TCGA RNA-seq dataset.
