# Supplementary material for: Tumor Cell Extrinsic Synaptogyrin 3 Expression as a Diagnostic and Prognostic Biomarker in Head and Neck Cancer
Source: Cancer Res Commun. 2022 Sep 15;2(9):987–1004. doi: 10.1158/2767-9764.CRC-21-0135 (PMC9491693; doi:10.1158/2767-9764.CRC-21-0135)
Supplement: Figure S2 — Validation of upregulated and downregulated genes in HPV(+) HNSC patient tumors. [file crc-21-0135-s02.docx]

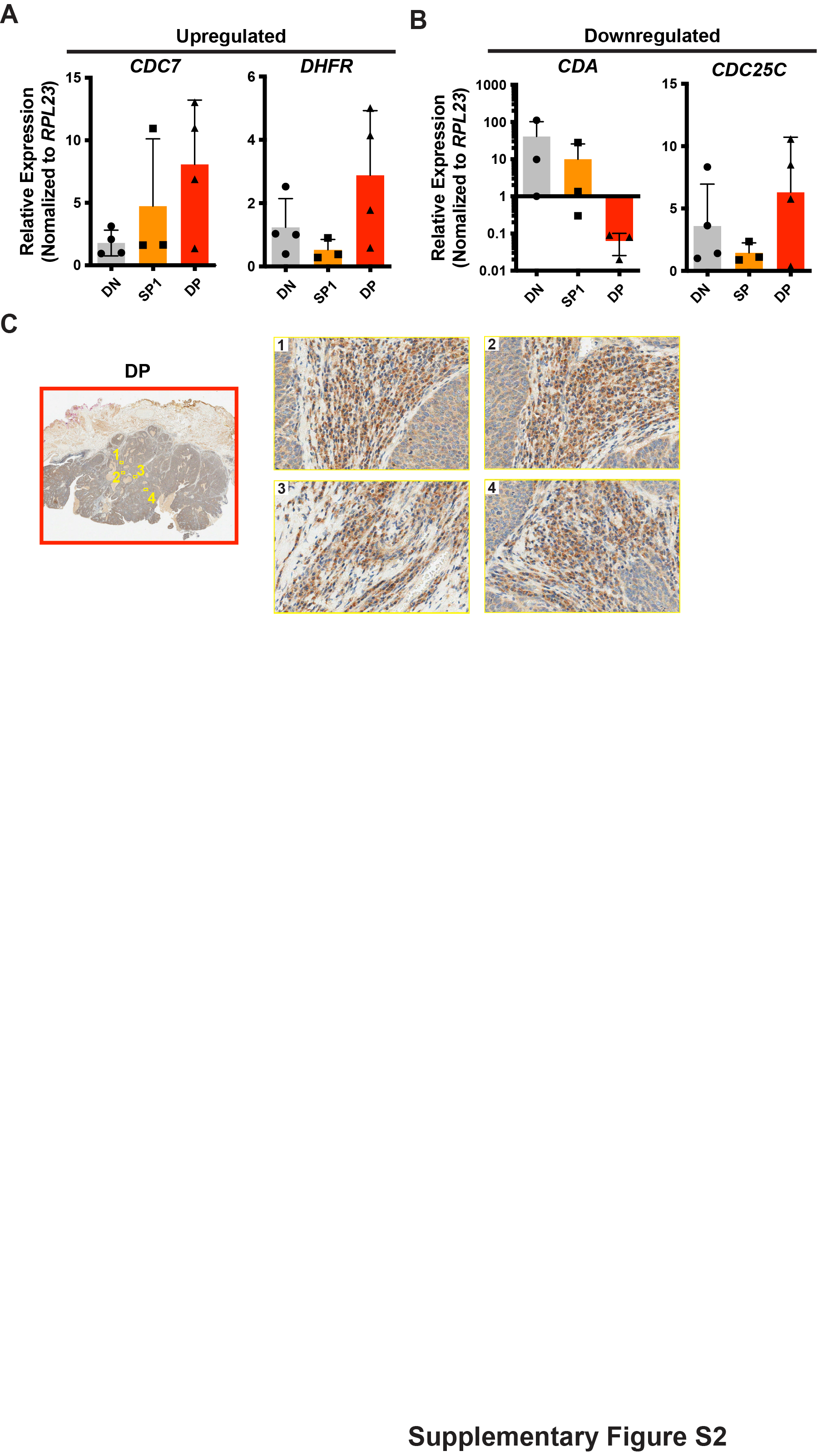


**Supplementary Figure S2. Validation of upregulated and downregulated genes in HPV(+) HNSC patient tumors. Related to Figure 2.**

Quantitative real-time PCR analysis of qPCR validation of genes found to be upregulated (A) or downregulated (B) in TCGA HNSC RNA-seq data using fresh frozen whole human HNSC patient tumors. Target gene expression was normalized to *RPL23* mRNA levels and fold expression calculated relative to the average of the DN group. Data are presented as the mean ± SEM (*n* = 4 technical replicates).

1. Analysis of SYNGR3 protein expression in the fresh frozen tumor validation cohort. Representative 1x and high magnification 20x inset images from several independent ROIs of SYNGR3 IHC staining within the tumor stroma according to each respective HPV assay category.
