# Supplementary material for: Tumor Cell Extrinsic Synaptogyrin 3 Expression as a Diagnostic and Prognostic Biomarker in Head and Neck Cancer
Source: Cancer Res Commun. 2022 Sep 15;2(9):987–1004. doi: 10.1158/2767-9764.CRC-21-0135 (PMC9491693; doi:10.1158/2767-9764.CRC-21-0135)
Supplement: Figure S3 — Additional multiplex staining coexpression and ROI quantification. [file crc-21-0135-s03.docx]

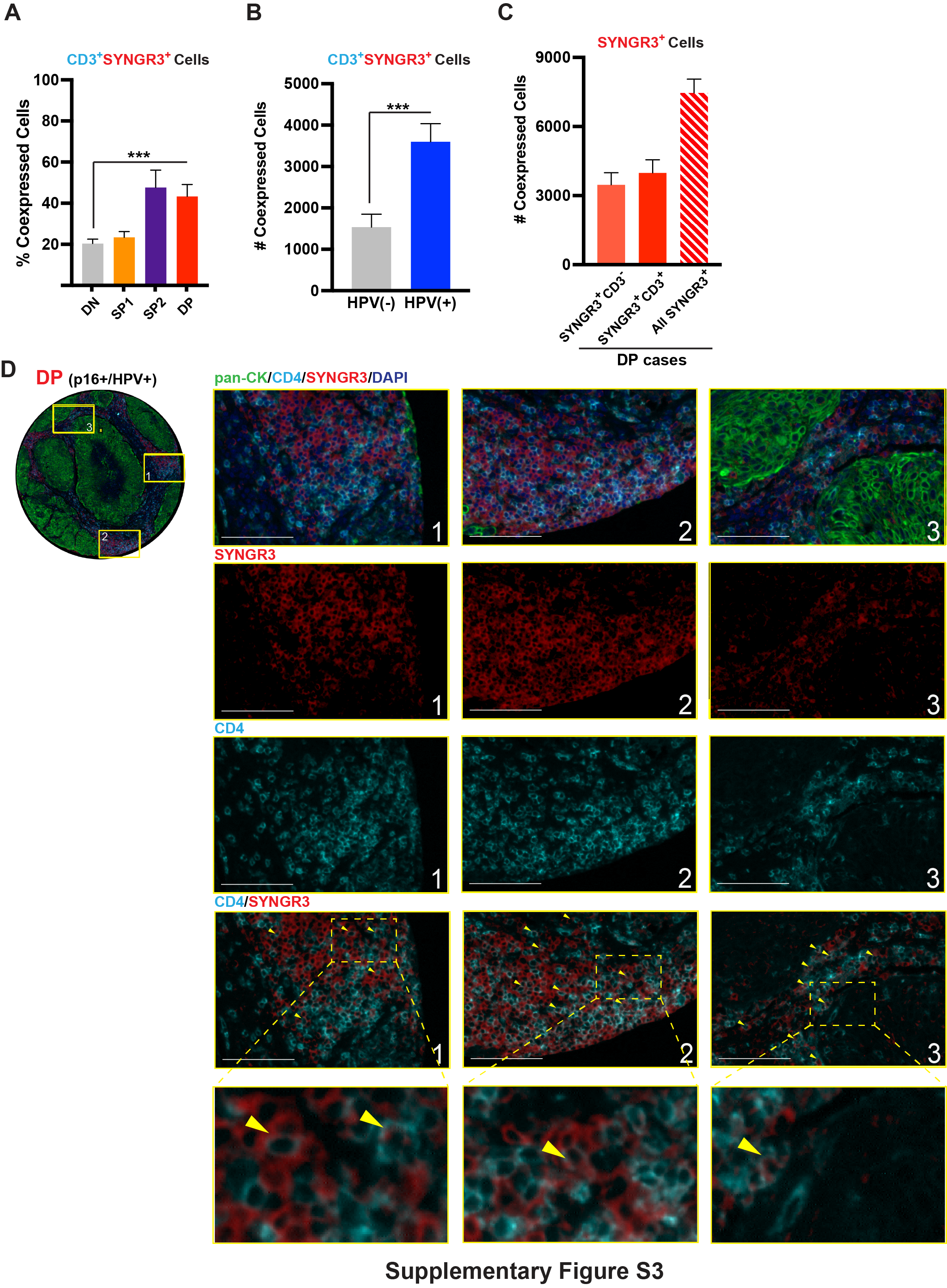


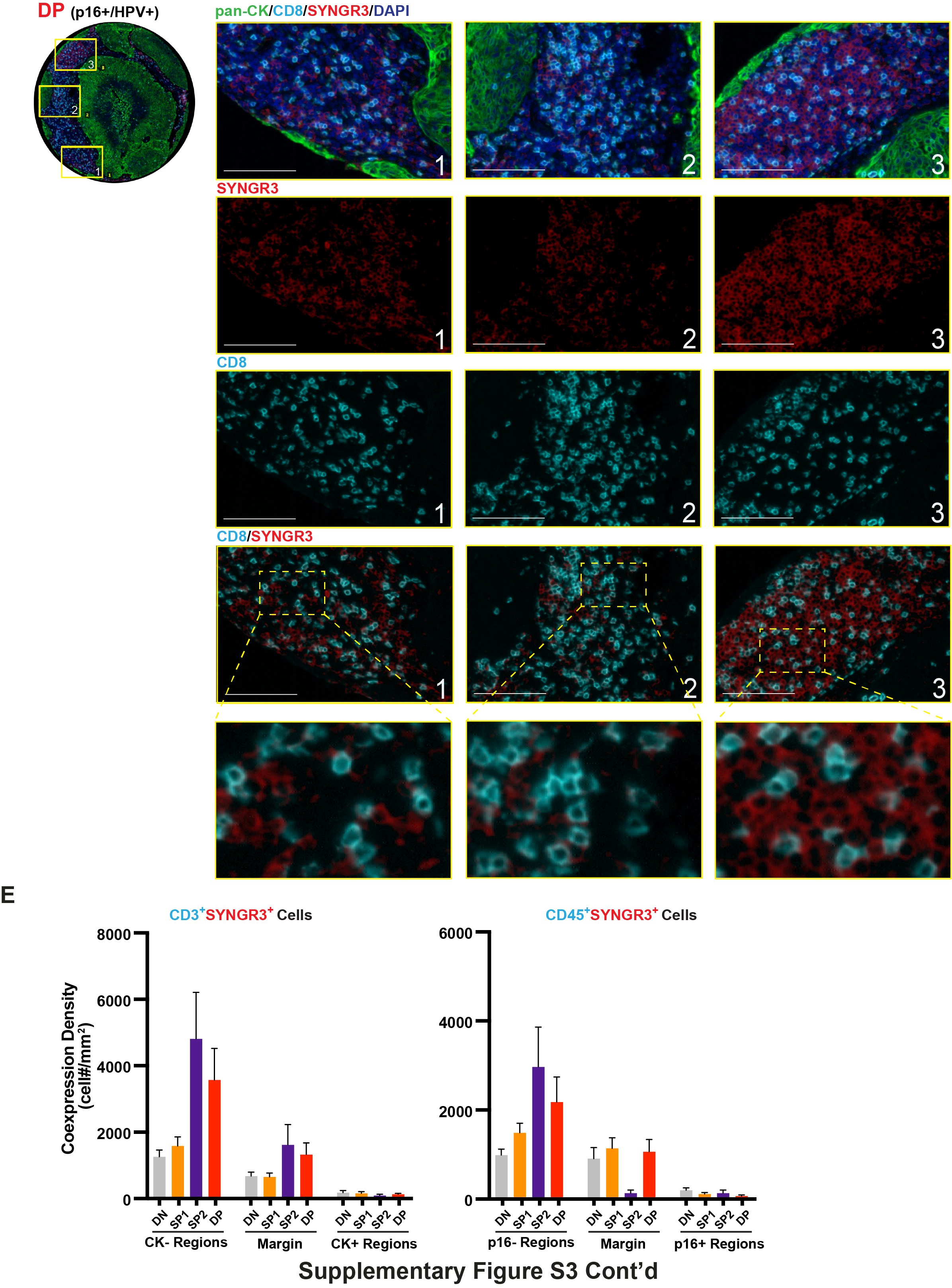


**Supplementary Figure S3. Additional multiplex staining coexpression and ROI quantification.**

1. Quantification of IHC multiplex staining from Figure 5A,C showing the percentage of total cells coexpressing SYNGR3 and CD3 (pan T cell marker) according to the different HPV assay categories in all regions of interest analyzed. Expression data are presented as mean ± SEM (*** *P* < 0.001).
2. Quantification of IHC multiplex staining of number of cells with SYNGR3 and CD3 (pan T cell) coexpression in samples with known HPV status established by ddPCR. Expression data are presented as mean ± SEM (*** *P* < 0.001).
3. Quantification of IHC multiplex staining showing number of cells expressing SYNGR3 within DP TMA cores, and highlighting cells with and without CD3 coexpression.
4. *Top*, representative multiplex IHC staining for SYNGR3 (red), CD4 (cyan, T helper cells), pan-CK (green, pan-cytokeratin), and DAPI (purple, nuclei) with dual positive SYNGR3+/CD4+ cells highlighted by yellow arrowheads in CK- regions.

*Bottom (D - cont’d)*, representative multiplex IHC staining for SYNGR3 (red), CD8 (cyan, cytotoxic T cells), pan-CK (green, pan-cytokeratin), and DAPI (purple, nuclei). Scale bar = 400 µm.

1. Quantification of IHC of SYNGR3 and CD3 (T cells; *left*) or SYNGR3 and CD45 (all immune cells; *right*) according to HPV assay category and separated by epithelial/stromal regions of interest. Epithelial region defined by either pan-CK (*left*) or p16 IHC (*right*), and separating out the tumor margin (defined by 25μM on either side of tumor border). Data represented as density of coexpressing cells. Expression data are presented as mean ± SEM.
