# Supplementary material for: Tumor Cell Extrinsic Synaptogyrin 3 Expression as a Diagnostic and Prognostic Biomarker in Head and Neck Cancer
Source: Cancer Res Commun. 2022 Sep 15;2(9):987–1004. doi: 10.1158/2767-9764.CRC-21-0135 (PMC9491693; doi:10.1158/2767-9764.CRC-21-0135)
Supplement: Figure S4 — Association of SYNGR3 expression patient survival. [file crc-21-0135-s04.docx]

**
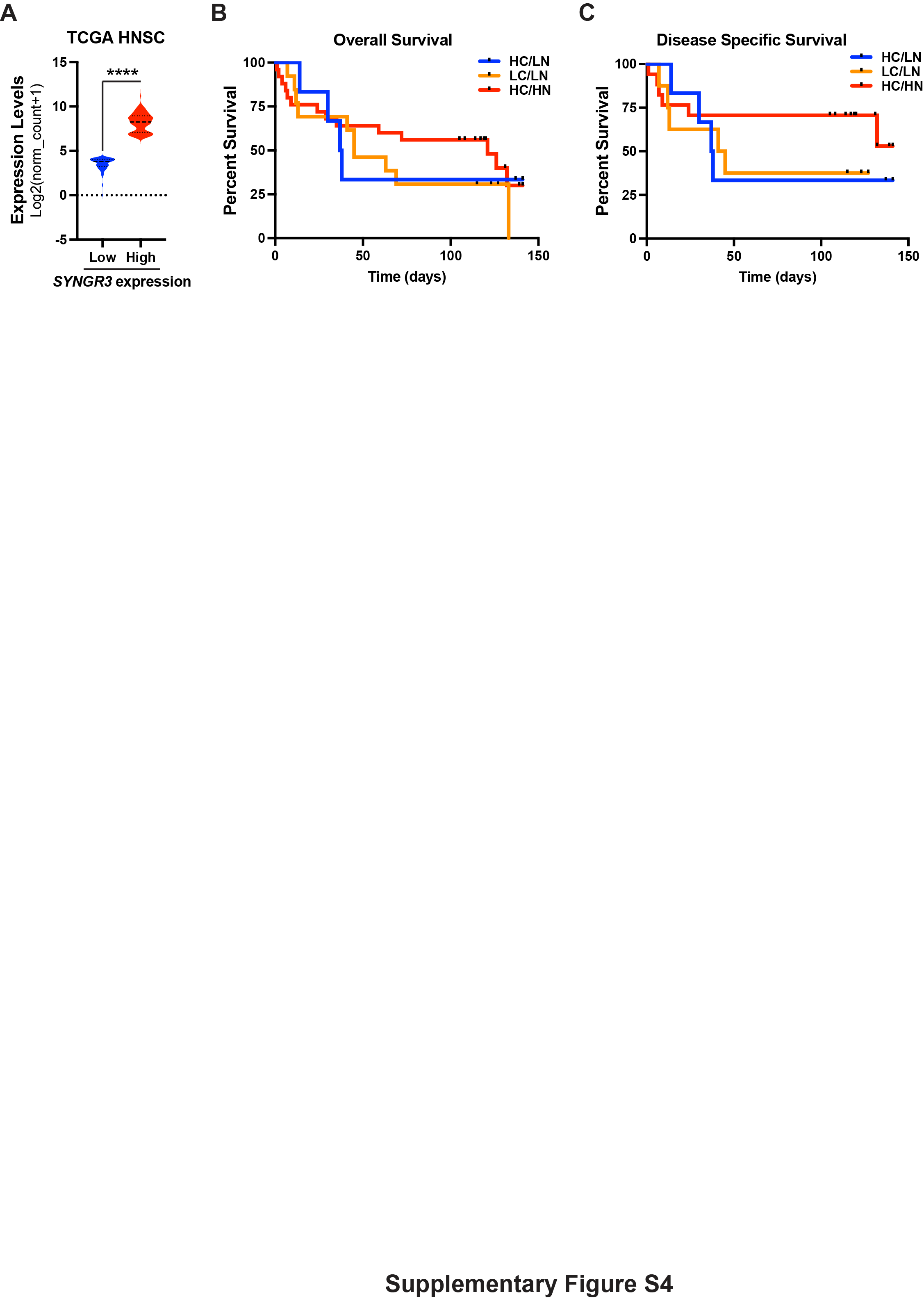
**

**Supplementary Figure S4. Association of *SYNGR3* expression patient survival.**

1. *SYNGR3* mRNA levels from TCGA HNSC patients stratified according to top and bottom quartile using UCSC Xenabrowser. High *SYNGR3* = top quartile mRNA expression, Low *SYNGR 3*= bottom quartile mRNA expression. (**** *P* < 0.0001).

(B-C) Survival curves of HPV(+) CHANCE TMA patients. Patients stratified by p16 cytoplasmic and nuclear expression by p16 IHC into localization categories. High cytoplasmic (HC) = cytoplasmic H-score of 50 and above; low cytoplasmic (LC) = cytoplasmic H score below 50; high nuclear (HN) = nuclear H-score of 70 and above; low nuclear (LN) = nuclear H-score below 70.
